# Remimazolam for Procedural Sedation During Computed Tomography Angiography in a Patient With Poor Myocardial Performance Due to Cardiomyopathy

**DOI:** 10.14740/jmc5371

**Published:** 2026-07-28

**Authors:** Joel Ravichandar, Kevin Spellman, Chris McKee, Joseph D. Tobias

**Affiliations:** a Northeast Ohio College of Medicine, Rootstown, OH, USA; b Department of Anesthesiology and Pain Medicine, Nationwide Children’s Hospital, Columbus, OH, USA; c Department of Anesthesiology and Pain Medicine, The Ohio State University College of Medicine, Columbus, OH, USA

**Keywords:** Remimazolam, Angiography, Procedural sedation, Benzodiazepine, Cardiomyopathy

## Abstract

Given their cognitive and developmental status, sedation is frequently required for procedural imaging in infants and children. Although generally free of adverse effects, the agents used for procedural sedation may impact airway, hemodynamic, and respiratory function, especially in the presence of comorbid cardiac involvement. We present the use of the novel benzodiazepine, remimazolam, administered by continuous infusion and supplemented with intermittent bolus doses of ketamine to provide sedation during computed tomography angiography in a 16-month-old girl, who presented with myocardial dysfunction related to a primary cardiomyopathy. Previous reports of remimazolam administration to patients with underlying comorbid cardiac disease are reviewed, and its applications in this scenario are discussed.

## Introduction

Remimazolam is a novel, ultrashort-acting intravenous (IV) benzodiazepine that produces sedation, anxiolysis, and amnesia through agonism of the γ-aminobutyric acid type A receptor (GABA_A_) [1, 2]. Its pharmacokinetic profile differs significantly from other benzodiazepines, as it undergoes rapid metabolism by nonspecific tissue esterases, resulting in a short elimination half-life of approximately 5–10 min and a limited context-sensitive half-life. These characteristics allow for a rapid onset and offset of its clinical effects, predictable recovery, and minimal drug accumulation even with prolonged infusions. Following its approval by the United States Food and Drug Administration (FDA) for procedural sedation in adults, remimazolam has been increasingly utilized as both a primary sedative agent and as an adjunct to general anesthesia in pediatric-aged patients [3]. Early clinical trials have demonstrated its efficacy in a variety of procedural settings, including gastrointestinal endoscopy and flexible bronchoscopy, with a favorable safety profile [4–6]. Compared to commonly used agents such as propofol, remimazolam has been associated with fewer adverse hemodynamic effects, including reduced rates of hypotension during procedural sedation and induction of general anesthesia [4, 5, 7].

In pediatric patients undergoing imaging studies, sedation is often necessary to achieve adequate image quality due to the need for complete immobility, especially in patients with limited ability to cooperate given their cognitive level of development [8–10]. However, the selection of an appropriate sedative agent can be challenging in patients with underlying cardiac disease, as many commonly used agents may have negative inotropic or chronotropic effects. Agents such as propofol and dexmedetomidine may lead to hypotension or bradycardia, potentially exacerbating underlying cardiovascular dysfunction. In contrast, preliminary clinical evidence suggests that remimazolam may provide effective sedation with minimal impact on hemodynamic stability, making it an attractive alternative in this high-risk population [10, 11].

Despite growing evidence in adult populations, data regarding the use of remimazolam in children with significant comorbid conditions remain limited [3]. We present our anecdotal experience with the use of remimazolam and ketamine for procedural sedation during computed tomography (CT) angiography in a pediatric patient with depressed myocardial function secondary to cardiomyopathy.

## Case Report

This retrospective review was approved by the Institutional Review Board of Nationwide Children’s Hospital (Columbus, Ohio). This study was conducted in compliance with the ethical standards of Nationwide Children’s Hospital for research involving human subjects, as well as with the Helsinki Declaration.

A 16-month-old, 10.5-kg female toddler with no significant past medical history presented to the emergency department with several days of fever, cough, respiratory distress, and progressive lethargy. Initial evaluation revealed substantial cardiomegaly on chest radiograph. Echocardiography demonstrated severe dilation of the left ventricle with severely diminished systolic function (ejection fraction approximately 10%). She was admitted for further evaluation and management of acute heart failure related to dilated cardiomyopathy. On presentation, vital signs were notable for a heart rate (HR) of 160 beats/min, blood pressure (BP) of 73/50 mm Hg, respiratory rate of 26 breaths/min, oxygen saturation of 97% on room air, and a temperature of 37.7 °C. Cardiovascular examination was notable for a gallop rhythm with an S_3_ without murmur and capillary refill of less than 2 s. Laboratory evaluation was notable for a brain natriuretic peptide level of 4,828 pg/mL and an elevated troponin I of 0.302 ng/mL. Additionally, a mild leukocytosis (white blood cell (WBC) 19.0 ×10^3^/µL) and anemia (hemoglobin 9.8 g/dL) were present. Other laboratory values, including electrolytes, renal function, liver function tests, and lactate, were within normal limits.

During a previous anesthetic 5 days earlier for placement of a percutaneous IV central catheter (PICC), hypotension occurred following the administration of midazolam (1 mg), ketamine (total of 30 mg in three divided doses), and propofol (2 mg), which required the administration of a single dose of epinephrine (5 µg). Given concern for a potential structural etiology for the cardiomyopathy, including anomalous left coronary artery from the pulmonary artery (ALCAPA), computed tomography (CT) angiography was planned for further evaluation of the coronary anatomy.

At the time of the procedure, the patient was receiving continuous infusions of milrinone (0.5 µg/kg/min) and epinephrine (0.02 µg/kg/min). Additional medications included chlorothiazide (10 mg/kg IV every 12 h), furosemide (10 mg IV every 6 h), and spironolactone (1 mg/kg/day by mouth). Pre-procedure examination demonstrated an awake patient with a stable hemodynamic status and no respiratory distress. Vital signs revealed a HR of 130 beats/min, BP of 88/42 mm Hg, and oxygen saturation of 97% on room air. Airway examination was unremarkable. Breath sounds were clear bilaterally, and cardiovascular findings were unchanged from earlier examinations. The patient was assigned an American Society of Anesthesiologists (ASA) physical status 5 given her severely depressed myocardial function and requirement for inotropic support. The patient was held *nil per os* for 6 h while maintenance IV fluids were provided through an IV cannula. The patient was transported to the imaging suite where routine ASA monitors were placed. Supplemental oxygen was administered and end-tidal carbon dioxide (ETCO_2_) was monitored from a nasal cannula. Following application of standard ASA monitors, remimazolam was initiated at 20 µg/kg/min and then increased to 30 µg/kg/min after 15 min. Sedation was supplemented with a total of 15 mg of ketamine, administered in divided doses of 5 mg (0.5 mg/kg). Toward the end of the procedure, the remimazolam infusion was decreased to 20 µg/kg/min and then discontinued. During the 45–60 min procedure, the patient maintained a patent airway with spontaneous ventilation with an oxygen saturation of 97–99%, an ETCO_2_ of 25–31 mm Hg measured via nasal cannula, and a respiratory rate of 20–30 breaths/min. BP ranged from 75–90/40–60 mm Hg with a HR of 120–150 beats/min. There was no need to escalate vasoactive support. The CT angiography was completed successfully without interruption or the need for conversion to general anesthesia. No coronary artery anomalies were noted on the angiography. Following the procedure, the patient was transported back to the cardiothoracic intensive care unit in stable condition. The epinephrine and milrinone infusions were weaned as there was recovery of hemodynamic function. The patient was transitioned to an oral medication regimen for congestive heart failure including enalapril (2 mg twice a day), spironolactone (1.25 mg/kg once a day), carvedilol (0.5 mg/kg twice a day), dapagliflozin (1.25 mg once a day), furosemide (1 mg/kg twice a day), and aspirin (40.5 mg once a day). She was discharged home after a 2-week hospitalization and continues regular follow-up with the pediatric cardiology service.

## Discussion

Initial clinical experience with remimazolam in adults has demonstrated its efficacy and safety for procedural sedation and its emerging role as an adjunct to general anesthesia. Its use has been most extensively described in endoscopic and other minimally invasive procedures, where it provides reliable sedation with a rapid onset and favorable recovery profile [4–6]. Compared to propofol, remimazolam may be associated with fewer adverse hemodynamic effects including a lower incidence of hypotension during procedural sedation and induction of general anesthesia [4, 5, 7]. When compared to midazolam, remimazolam offers a shorter half-life, smaller volume of distribution, and thus shorter context sensitive half time offering a quicker recovery. These characteristics have led to increasing interest in its broader application within adult anesthesia practice, including its use for the induction and maintenance of general anesthesia.

In contrast to the adult population, the clinical experience with remimazolam in pediatric patients remains more limited, with the majority of available data restricted to anecdotal case reports and small case series [3]. Similar to the adult population, these anecdotal reports have described the efficacy of remimazolam in various clinical scenarios including both invasive and noninvasive procedures [3, 9]. However, data regarding its use in infants and young children, particularly those with significant comorbid conditions, including underlying cardiac disease, remain sparse.

Data from the adult population have demonstrated potential advantages of remimazolam use in patients with comorbid cardiovascular diseases. In a prospective, randomized, double-blind trial of 60 adult patients undergoing mitral, aortic, or double-valve replacement surgery using cardiopulmonary bypass, Liu et al compared the induction of anesthesia with remimazolam (0.3 mg/kg, n = 30) to propofol administered by target-controlled infusion to achieve a serum concentration of 2.5 µg/mL (n = 30) [12]. Remimazolam was associated with a lower incidence of hypotension, less fluctuation in mean arterial pressure, and lower cumulative norepinephrine requirements during induction when compared with propofol. In patients undergoing cardiac ablation for atrial fibrillation under general anesthesia, remimazolam was associated with significantly reduced vasoactive agent requirements and improved hemodynamic stability when compared with desflurane [13]. Similarly, in 20 elderly patients (median age 84 years) with severe aortic stenosis, remimazolam combined with remifentanil proved to be a safe and effective agent for the induction of anesthesia, with the need for limited pharmacologic support of mean arterial pressure with either ephedrine or phenylephrine [14]. In a single-center, prospective, randomized, double-blinded trial of 117 adult patients undergoing valve replacement surgery, Hu et al compared low-dose remimazolam (0.2 mg/kg, n = 39), high-dose remimazolam (0.3 mg/kg, n = 39), and etomidate (1.5 mg/kg, n = 39) for anesthetic induction [15]. Low-dose remimazolam provided hemodynamic stability comparable to etomidate, while both low-dose remimazolam and etomidate were associated with less mean arterial pressure fluctuation, a lower incidence of hypotension, and lower cumulative norepinephrine requirements than high-dose remimazolam. Both the high-dose and low-dose of remimazolam regimens were associated with fewer incidences of myoclonus and injection pain compared to etomidate.

Despite the supporting evidence in adult patients, the data regarding remimazolam use in pediatric patients with comorbid cardiac disease are primarily limited to anecdotal case reports or small case series. Hosokawa et al retrospectively reviewed the use of remimazolam in 39 pediatric patients, ranging from 2 months to 16 years, during cardiac catheterization [16]. The median remimazolam dose required for loss of consciousness was 0.34 mg/kg with a mean maintenance infusion rate of 1.0 mg/kg/h and a median recovery time of 15 min. Although 15 patients had a 30% decrease in BP and eight patients required the bolus administration of a vasoactive, no life-threatening adverse events were reported, and the authors ultimately concluded that remimazolam was a reasonable alternative anesthetic agent in pediatric patients undergoing cardiac catheterization. In a prospective trial, Jin et al investigated the median effective dose (ED_50_) and 95% effective dose (ED_95_) of remimazolam for preoperative sedation in 78 children, 1 month to 6 years of age, with acyanotic congenital heart disease (CHD) [17]. The ED_50_ values for successful sedation were 0.209 mg/kg in infants, 0.259 mg/kg in toddlers, and 0.266 mg/kg in preschool-aged children, while the corresponding ED_95_ values were 0.356, 0.404, and 0.408 mg/kg, respectively. No serious adverse effects were observed with IV remimazolam, supporting its potential role for preoperative sedation in children with CHD. Li et al evaluated the hemodynamic effects of a single IV dose of remimazolam in 35 children (median age 6.67 years) with CHD undergoing cardiac catheterization [18]. Following a bolus dose of remimazolam (0.3 mg/kg), the authors reported no significant change in HR, mean arterial pressure, cardiac output, cardiac index, right atrial pressure, or pulmonary artery pressure, further supporting the hemodynamic stability of remimazolam in pediatric patients with CHD. Anecdotal experience has also demonstrated the safety of remimazolam in patients with cardiac conduction abnormalities and for sedation during cardioversion [10, 19].

In our patient, remimazolam was used as the primary sedative agent in a 16-month-old child with newly diagnosed dilated cardiomyopathy. The patient was at risk for adverse effects during procedural sedation given the presence of severely depressed left ventricular dysfunction, ongoing requirement for inotropic support, and a prior history of recent hemodynamic instability during an earlier sedation. Sedation with remimazolam, supplemented by small doses of ketamine (0.5 mg/kg), allowed for completion of the coronary angiography over 45 min while maintaining spontaneous ventilation, adequate oxygenation, and near baseline hemodynamic status. Although our patient was receiving cardiovascular support with epinephrine and milrinone which may have mitigated the impact of sedation with remimazolam and ketamine, there was no need for escalation of vasoactive support, airway intervention, or conversion to general anesthesia. Although anecdotal, this experience supports the potential role of remimazolam as an ideal sedative option in pediatric patients with impaired myocardial performance.

Published pediatric reports suggest that remimazolam dosing varies according to the intended use, with bolus dosing described for preoperative sedation or induction/loss of consciousness and infusion dosing described for maintenance of anesthesia or procedural sedation [16–18]. In our patient, sedation was initiated with a bolus dose of ketamine and then maintained by remimazolam, administered as a continuous infusion at 20 µg/kg/min without a bolus dose. The infusion was subsequently increased to 30 µg/kg/min to maintain the desired level of sedation. This infusion rate (20–30 µg/kg/min) corresponds to 1.2–1.8 mg/kg/h, allowing comparison with pediatric reports describing maintenance infusion rates near 1.0 mg/kg/h [16]. In the setting of severe myocardial dysfunction and prior hypotension during sedation, this approach avoided administration of other sedative agents which would be more likely to compromise hemodynamics or respiratory status.

### Learning points

Remimazolam is a novel, ultrashort-acting benzodiazepine that acts as a GABA_A_ receptor agonist, producing amnesia, sedation, and anxiolysis. Although not currently FDA-approved for use in pediatric patients, clinical experience has demonstrated its efficacy as a primary or adjunctive agent for sedation and general anesthesia in the pediatric population. Remimazolam undergoes rapid metabolism by tissue esterases, resulting in a short context-sensitive half-life and predictable recovery profile. When compared with propofol, it has been shown to have a more favorable hemodynamic profile with lower rates of hypotension, supporting its use in vulnerable populations. Regardless of the agents used for procedural sedation, standard ASA monitoring, supplemental oxygen, ETCO_2_ monitoring, reliable IV access, and immediate availability of vasoactive medications and airway equipment remain essential when providing sedation in high-risk pediatric patients.

## Data Availability

Any inquiries regarding supporting data availability of this study should be directed to the corresponding author.
